# The experiences of physiotherapy independent prescribing in primary care: implications for practice

**DOI:** 10.1017/S1463423623000142

**Published:** 2023-04-20

**Authors:** Jacqueline Mullan, Janet Smithson, Nicola Walsh

**Affiliations:** 1 University of the West of England, Blackberry Hill, Stapleton, Bristol, UK; 2 University of Exeter, Stocker Road, Exeter, UK

**Keywords:** physiotherapy independent prescribing, primary care

## Abstract

**Aim:**

To explore the experiences of musculoskeletal (MSk) physiotherapy independent prescribing in primary care from the perspectives of physiotherapists and General Practitioners (GPs) and identify the implications these have for contemporary physiotherapy practice in primary care.

**Background:**

Legislative change in the United Kingdom (UK) in 2013 enabled physiotherapists holding a postgraduate non-medicalprescribing qualification to independently prescribe certain drugs that assist in patient management. Independent prescribing by physiotherapists is a relatively contemporary development in role change and purpose, occurring alongside the development of physiotherapy first contact practitioner (FCP) roles in primary care.

**Methods:**

A critical realist approach was used, with qualitative data collected via 15 semi-structured interviews with physiotherapists and GPs in primary care. Thematic analysis was applied.

**Participants:**

Fifteen participants were interviewed (13 physiotherapists, 2 GPs). Of the 13 physiotherapists, 8 were physiotherapy independent prescribers, 3 were MSk service leads, and 3 were physiotherapy consultants. Participants worked across 15 sites and 12 organisations.

**Findings:**

Whilst physiotherapists were empowered by their independent prescribing qualification, they were frustrated by current UK Controlled Drugs legislation. Physiotherapists reported vulnerability, isolation, and risk as potential challenges to independent prescribing, but noted clinical experience and ‘patient mileage’ as vital to mitigate these. Participants identified the need to establish prescribing impact, particularly around difficult to measure aspects such as more holistic conversations and enhanced practice directly attributed to prescribing knowledge. GPs were supportive of physiotherapists prescribing.

**Conclusions:**

Establishment of physiotherapy independent prescribing value and impact is required to evaluate the role of, and requirement for, physiotherapy independent prescribers within primary care physiotherapy FCP roles. Additionally, there is a need for a review of physiotherapy prescribing permitted formulary, and development of support mechanisms for physiotherapists at individual and system levels to build prescribing self-efficacy and autonomy, and to advance and sustain physiotherapy independent prescribing in primary care.

## Background

Independent prescribing legislation for physiotherapists was established via a legislative change to the Human Medicines Regulation 2013 (Chartered Society of Physiotherapy (CSP), 2013). At the time, it was suggested by the Department of Health (DOH) that prescribing would “mean patients will no longer have to go back to their doctors to get medication after visiting the physiotherapist,… freeing up valuable time for General Practitioners (GPs) and making things more convenient for the patient” (DOH, 2013).

Since then, the wider health agenda has moved forward considerably, particularly in relation to focus and flexibility in the delivery of primary health care (National Health Service (NHS) England, 2014; NHS England, 2017; NHS England, 2019a, 2019b). GPs were described as overworked, overloaded, exhausted (Mathers, 2016), and under pressure (Lacobucci, 2019) with a proposed need for 5,000 more GPs and a team of multiple professionals in primary care to provide a flexible workforce to meet patient and population need (NHS England, 2014). Subsequently, the focus of healthcare has moved more overtly towards primary and community care with the publication of the NHS Long Term Plan (NHS England, 2019a) and development of primary care networks (PCNs) of local GP practices and community teams (NHS England, 2019a).

The resultant role opportunities such as first contact practitioner (FCP) (Goodwin and Hendrick, 2016; Moffatt, Goodwin and Hendrick, 2018), and advanced clinical practitioner (ACP), and their underpinning frameworks (Health Education England (HEE), 2017; Health Education England, NHS England and Skills for Health, 2018, HEE, 2020), require professionals such as physiotherapists to develop new knowledge, skills and competencies, and additional educational qualifications as part of the evolving scope and levels of practice. An example of such a postgraduate attribute and qualification for physiotherapists is non-medical prescribing to become an independent prescriber.

The focus of this research is physiotherapy independent prescribing in relation to MSk physiotherapists working in primary care.

As a new area of practice for physiotherapists, research findings have begun to emerge. These include case studies, personal reflections on prescribing journey, views on anticipated opportunities and barriers, and the role of legislative frameworks underpinning physiotherapy independent prescribing.

Loughran and Rae (2015) presented a specific case study on a usually active 26-year-old female presenting with lower back pain and neuropathic leg pain and concluded that prescribing by the physiotherapist enabled faster access to appropriate medicines and could be well integrated as part of specialist assessment and shared decision-making (Loughran and Rae, 2015).

Hey (2018) reflected on his evolution from supplementary prescriber (in 2006) to independent prescriber (in 2015) and beyond, noting the loneliness and isolation of being one of the first of his profession to move into this area: the need to take personal responsibility to engage in developing prescribing-specific governance processes and strategic workforce planning to support and enable others on the path to prescribing (Hey, 2018). As a coping mechanism for the additional responsibility, Cope, Tully and Hall (2019) found a clear link between prescribing self-efficacy and the willingness to take responsibility for prescribing decisions in their study of non-medical prescribers (nurses, pharmacists, and physiotherapists) on acute medical units in UK hospitals, albeit only 4 out of 99 participants were physiotherapists.

Whilst the UK has achieved independent prescribing rights for physiotherapists, Australia is still lobbying for those rights. Noblet *et al*. (2018), Noblet *et al.* (2019a) and Noblet *et al*. (2019b) undertook a multi-faceted study to gather prospective views of physiotherapists and physiotherapy students in relation to the potential implementation of physiotherapist prescribing in Australia. Anticipated benefits mainly focussed on improved delivery of health services (80.1%). Potential barriers included caution in relation to the level of responsibility being too great (43.9%), physiotherapists not having adequate pre-knowledge to train as a prescriber (34.1%), and increased safety risk to patients (34.1%) (Noblet *et al.*, 2019a). It is worth noting that unlike the current study, these views are prospective views and are not based on experience. Other barriers noted in the literature include frustration at the restrictions on certain controlled drugs in the UK, particularly if working in pain services or a FCP role (Hey, 2018), and some reticence around the extra responsibility related to patient safety and the “legal consequences in case of harm” that it brought (Holden *et al.*, 2019, p. 333).

In their systematic mixed study review in relation to medicine management activity with physiotherapy and podiatry, Stenner *et al.* (2018) noted that the UK legislation is helpful in establishing a framework and boundaries within which physiotherapists can work with clear educational, competency, and prescribing standards. The study suggests an area of ambiguity worldwide where physiotherapists are advising patients about medicines and are involved in administering medicines (both prescription and non-prescription) without clear underpinning medicines management processes or prescribing qualification (Stenner *et al.*, 2018), reflecting Kumar and Grimmer’s (2005) findings in relation to non-steroidal anti-inflammatory drugs and Holden *et al.*’s (2019) findings in the context of hip osteoarthritis.

This “identified a mismatch in many countries between client demand for medicines and medicines advice and the educational preparation and governance to support physiotherapists to meet this demand” (Stenner *et al.*, 2018, p. 1338). Stenner *et al.*’s (2018) conclusion recognises that whilst legislation is in place, the next stage for research in the UK is to both evaluate the impact of prescribing and explore the views of key stakeholders regarding the changes in policy related to prescribing practice. This latter view aligns with this current research question.

## Methodology

A qualitative research methodology was chosen with data collection via semi-structured interviews. This research used critical realism as a philosophical framework (Fletcher, 2017). Thus, a range of applied and theoretical literature informed the structure of the semi-structured interview questions alongside the findings of two expert reference groups, and a patient and public involvement (PPI) group, conducted during the development phase of the research.

### Recruitment

Recruitment of physiotherapists, physiotherapy service managers, and GPs was by local and national purposive sampling. The selection criteria were that participants had an interest in, or experience of, either physiotherapy independent prescribing and/or primary care musculoskeletal (MSk) services.

### Consent processes

Each participant was given an information sheet, and once they volunteered to participate, they were asked to provide informed consent (either in writing via consent form or via recorded audio consent) and complete a participant demographics form.

### Interview schedule

Semi-structured interview schedules related to each of the three main groups of participants were designed in line with Health Research Authority (HRA) Approval requirements. (HRA Integrated Research Application System (IRAS) project number 238 300: protocol number 1718/29). The interview schedules were similar in overall content but had context and language specific to physiotherapists, physiotherapy service leads or GPs.

### Data collection and analysis

Interviews were face-to-face, via telephone or via Skype, and were recorded and transcribed. NVIVO was utilised as a tool for coding the data and managing the analysis (Jackson and Bazeley, 2019). This coding process was primarily via theory-driven code book deductive coding to enable comparison of the findings to prior research to support, extend or challenge previous findings (Boyatzis, 1998), and followed up by inductive coding to identify new aspects from the data and give balance. Thematic analysis within a critical realist framework was used to develop themes and create a journey of discovery (Terry *et al.*, 2017), with experiences and meaning being examined primarily at a semantic level and secondarily, at a latent level (Boyatzis, 1998; Terry *et al.*, 2017; Willig and Stainton Rogers, 2017).

## Findings

In total, 15 semi-structured interviews took place (excluding the two pilot interviews) with participants from a range of professional and managerial roles who all had in common an interest in physiotherapy independent prescribing or MSk services in primary care. Interviews ranged from 35 to 70 mins duration. The data collection was undertaken between October 2018 and May 2019 at a time of relative novelty for physiotherapy FCP services and roles, and also physiotherapy independent prescribing within them (Table 1).

**Table 1.** Overview of the background of participants

Descriptor of background of the participants	Number of participants meeting this description	Participant number
Physiotherapy independent prescribers with a minimum of 6 months of experience working in primary care within musculoskeletal (MSk) services	Eight	Participants 3,7,8,9,10,11, 12,15
Physiotherapists who were not independent prescribers but who worked in primary care within MSk services	Five	Participants 1,2,4,5,13
Consultant physiotherapists working within MSk services in the UK	Three	Participants 1,2,3
Managers of MSk community/primary care physiotherapy services	Three	Participants 1,3,13
GPs with an interest in MSk services or commissioning	Two	Participants 6,14
Commissioners for MSk primary care healthcare	Two	Participants 5,6

A key has been used to indicate the designations of participants.

P1 = Participant 1; P2 = Participant 2 etc.

PT = Physiotherapist; GP = General Practitioner.

IP = Independent Prescriber; Not IP = Not an Independent Prescriber.

In reviewing the data about the experiences of physiotherapy independent prescribing in MSk primary care, the analysis focussed on the aspects specific to the implications for practice. Two themes, each with subthemes, were identified (Table 2).

**Table 2.** Themes and subthemes

Theme	Subthemes
1. Adaptability and responsibility: pioneering physiotherapy independent prescribing	• Early adopters as pioneers • Vulnerability and risk • Resilience and sustainability
2. The Unexpected Impact: of becoming aphysiotherapy independent prescriber	• More focussed conversations leading to enhanced practice • Deprescribing

## Theme 1: adaptability and responsibility: pioneering physiotherapy independent prescribing

### Theme 1.1: early adopters as pioneers

A sense of personal and professional responsibility to get it right for future generations of physiotherapists and patients was articulated about physiotherapy independent prescribing particularly within FCP roles.

*I enjoy feeling as if I’m at the fighting edge… of physiotherapy and the changes that are going ahead. (P12/PT/IP)*

Frustration emerged around autonomy and ability to make their own decisions due to covert and overt external constraints (Prescribing Information Technology (IT) access, permitted physiotherapy prescribing formulary, Controlled Drugs legislation). Physiotherapy independent prescribing restrictions regarding Codeine, Tramadol, Gabapentin, and Pregabolin were particularly noted as impactful in the MSk primary care setting.

*I probably prescribe less than I thought I would but I think some of that is hampered by our formulary at the moment, especially in MSk medicine with the Controlled Drugs that we do have available, and the loss of Gabapentin and Pregabalin, in my opinion, is absolutely a step backwards. (P12/PT/IP)*

*Oh, it’s just this whole, ridiculous, Controlled Drugs thing. It’s just nonsensical that you can prescribe liquid Morphine and Dihydrocodeine, but you can’t prescribe Codeine and Tramadol. (P10/PT/IP)*

### Theme 1.2: vulnerability and risk

The reality of translating physiotherapy prescribing into practice was highlighted.

*I think the biggest thing for me is about the transition from going to a course, doing a course and actually, how do you do this. So, it’s not just about the understanding, but the logistics…actually how do we make this happen in primary care? (P11/PT/IP)*

Vulnerability was directly linked to the risk-taking associated with the independent prescribing and influenced by the level of support and governance processes in place in individual organisations.

*I think the position you put yourself in is quite at risk: you have an awful lot of responsibility to take on board when it comes to screening patients… I think we are more vulnerable (P7/PT/IP)*

Clinical experience and ‘patient mileage’ were directly associated with reported confidence in prescribing ability and decision-making.

*Well I think you need to be at a level, in terms of clinical reasoning, that you are going to cope. Because it’s really stressful and you are far more vulnerable than in any other job role I’ve ever been in before, in terms of your professional governance. (P10/PT/IP)*

*The bit again which is difficult to quantify is patient mileage, is pattern recognition and perhaps being able to read between the lines in consultation. (P8/PT/IP)*

### Theme 1.3: resilience and sustainability

The sustainability of physiotherapy independent prescribing within primary care was multifaceted: building resilience in individuals; creating a sustainable physiotherapy career pathway and staff development pipeline; establishing support networks; managing workloads; and promoting physiotherapy independent prescribing beyond the physiotherapy profession were all identified as vital.

The resilience aspect was related to being more comfortable in their own ability, feeling less vulnerable, and more able to take risks.

*The thing we’ve learned the hard way is, if you send somebody on prescribing too soon and they are not a good diagnostician, they are not a good prescriber. (P3/PT/IP)*

The complexity of sustainability of physiotherapy independent prescribing was summarised in the contemporary context of the evolving FCP practice situation.

*I think probably at the moment the thing needs to be in bringing people up to this skill level and perhaps backfilling physio departments. I feel that we have absolutely asset-stripped some of our physio departments at the moment of all the senior clinical specialist level staff and it’s going to take a number of years for that void to be filled. So, I think getting people into first contact roles is absolutely great, get them established and as things change with the formulary then start to look at releasing to prescribe at that time. (P12/PT/IP)*

The sustainability of physiotherapy independent prescribing in MSk primary care was also directly linked to the promotion of it beyond the physiotherapy profession. Neither of the General Practitioner participants (P6, P14) was aware of physiotherapy prescribing as a potential.

*I’ve never even heard about it [physiotherapists prescribing] until this. (P6/GP/IP)*

Linked with a discussion about what the priorities for physiotherapy in MSk primary care services are, there were mixed views from the two GP participants.

*I can see there’s a huge potential there because as we keep reading every day, GPs are completely snowed under, so any help would be gratefully received…I’ve always been extremely impressed by the standard of physios that I have come across. I think the profession is very highly trained and probably underutilised in many respects. I see them [prescribing, injecting etc] as a very good use of your skills or an additional use of your skills more than anybody really. (P14/GP/IP)*

*I would rather that they [physiotherapists] a) be able to treat, b) order the appropriate investigations, c) access to the psychological therapy part of things – I think that’s really important, smoking cessation, weight, and diet. That’s more important to me than being able to prescribe. If they could do that, I’m happy to prescribe. If they want to prescribe, fine, on a limited set of drugs, whatever. But if I were to employ somebody, that is what I would be more interested in, because that is actually population changing … changing people’s quality of life, then life expectancy. (P6/GP/IP)*

All the physiotherapy participants reported positivity from GPs with whom they were working regarding physiotherapy independent prescribing. This is reflected in the following view.

*The only response [I’ve] had is a very positive one … we can manage the patients more completely without having to bother them [the GPs], effectively. (P8/PT/IP)*

Interestingly, the majority of physiotherapy participants had the view of prescribing specific to this primary care setting and FCP role as a valuable added extra rather than a necessity.

*Prescribing gives you another bow to your arrow … and I think sometimes it means that you can be more efficient in that one consultation, so you might save a patient having to go for a GP review or just be dealing with the patient there and then and they don’t have to come back to collect a prescription, etc. So, it makes you more efficient but I don’t think it’s the be-all and end-all. (P10/PT/IP)*

## Theme 2: the unexpected impact of becoming a physiotherapy independent prescriber

### Theme 2.1: more focussed conversations leading to enhanced practice

There was a strong view about how the knowledge and understanding gained through independent prescribing was being applied to the interaction with the patient (whilst not always resulting in a prescription) and how it was actually changing the physiotherapist/patient relationship and enhancing practice in a range of ways.

*I guess the thing I would say to people is it’s not the prescribing, it’s the knowledge of drugs, it’s the advice people get. So many people come to clinic and they have been in the system a long time … and they are still not using drugs appropriately, effectively. Or they are using too many of them. They often want a conversation about drugs …. And so I’ve used it [prescribing] an enormous amount there, on a better footing than ever conversations were had before. (P10/PT/IP)*

Participants who were prescribers reported more focussed and informed conversations that, in their view, enhanced holistic practice and optimised rehabilitation.

*So, all the time I think physios are explaining to patients that they need to use medication better to allow them to then do the rehab. For me, that’s the message. So, I think the whole holistic thing isn’t about not using medication at all, it’s about how do you use medication to allow the patient to go on a self-management journey. (P5/PT/Not IP)*

### Theme 2.2: deprescribing

Whilst the range of drugs actually prescribed by the physiotherapists in the MSk primary care setting was described by participants as fairly limited, a key reported role for physiotherapy independent prescribers was deprescribing, underpinned by a combination of confidence in having more focussed conversations and an ability to explore alternatives with patients.

*Deprescribing is something that we perhaps get involved with rather than prescribing and being able to step down medication because we have got other skills that we can offer a patient other than medication or investigation. (P12/PT/IP)*

*My first thought is keep people moving, public health,…So, it’s not suggesting they take something else but actually suggesting well if you are taking that still, do you think you need it? And I think previously I would have always stayed away from those conversations because I didn’t really understand what, necessarily, was going on and why they were taking those drugs. (P11/PT/IP)*

Deprescribing painkillers was seen as a particular remit for physiotherapists working in MSk primary care.

*So, painkillers, people get started on things and people forget. People have no idea why they are taking medications. (P3/PT/IP)*

Limitations of the deprescribing of some drugs were directly linked to the reported frustrations of prescribing, as physiotherapy independent prescribing constraints related to the same drugs.

## Discussion

This research explored the experiences and reality of physiotherapy independent prescribing particularly focussed on MSk health and primary care. The implications of physiotherapy independent prescribing on practice (service provision and patient care) were of specific interest. The findings in relation to the themes are discussed below.

Many of the physiotherapy practitioners in the primary care settings were being, or will be developed, into FCP roles, and there was an indication from this research that independent prescribing is a useful part of the toolbox of competencies and knowledge to underpin and enhance these roles. However, being a physiotherapy independent prescriber was not seen as essential by all participants, at least not in the initial stages of the FCP roles being established, being viewed instead as an additional attribute to enhance the coherence of practice.

The FCP role necessitates a different approach to traditional physiotherapy intervention. As articulated by participants, prescribing within this role requires considerable experience, advanced clinical reasoning, and a change in approach to their consultation to carry out appropriately and confidently. The uncertainty of the personal journey as an independent prescriber was evident reflecting Hey’s (2018) experiences of an ‘untrodden path’ (Hey, 2018, p159). Self-efficacy has been identified as a factor in influencing an individual’s competency to prescribe (Cope, Tully and Hall, 2019). Crucial to the achievement of potential prescribing self-efficacy in this research was confidence associated with clinical experience and ‘patient mileage’, which reflects Mandy, Saeter and Lucas’s (2004) positive correlation between length of time since qualification and general physiotherapy self-efficacy in Norwegian physiotherapists. Moreover, Moffatt, Goodwin and Hendrick (2018, p. 126) had highlighted physiotherapists’ view of the importance of clinical experience in enabling patients “to navigate their therapeutic journey in a more efficient manner”. Connected with improving self-efficacy and confidence was resilience building and avoidance of burnout, a challenge well recognised by GPs in primary care (Chambers, 1993; Kirwan and Armstrong, 1995; Soler, Yaman and Esteva, 2007; O’Dea *et al.*, 2017), with approximately 50% burnout risk being reported amongst UK GPs in 2015 (Staten and Lawson, 2017). Recognition and management of isolation, as also recognised by Noblet *et al.* (2019a), and vulnerability associated with the risk of prescribing were also identified as needing attention. Vulnerability was directly linked to the risk-taking associated with the independent prescribing and influenced by the level of support and governance processes in place in individual organisations, reflecting the findings of Dawson and Ghazi (2004) in relation to extended-scope practitioners. Supporting this, Holden *et al.* (2019) reported caution amongst physiotherapists regarding the extra responsibility and “legal consequence in case of harm” of prescribing (Holden *et al.*, 2019, p. 337).

Within a traditional target and clinical outcomes structure, justifying and evaluating physiotherapy independent prescribing within FCP roles was reported as a challenge, particularly as the actual prescribing rates were perceived as low. The biggest surprise for the physiotherapists was the “added extras” (or positive impacts) emerging as a result of the knowledge, competence, and confidence of becoming an independent prescriber. These were not directly related to the actual prescribing process but were nonetheless enhancing their practice.

Participants who were prescribers reported a greater sense of enhanced practice and increasingly focussed conversations that were more holistic and informed, particularly with patients but also with other practitioners. The more inclusive approach to healthcare was facilitated by the level of knowledge and holistic multi-system learning obtained from the prescribing postgraduate masters’ level programme. This had an impact beyond the physiotherapist being able to prescribe safely. These conversations were more complex than previously and facilitated a greater shared and informed management journey: embracing the patient-centred care and shared decision-making ethos promoted as a bedrock of the physiotherapy profession (CSP, 2020) and enhancing and underpinning the rehabilitation and self-care journeys of patients.

Demonstrating and measuring these indeterminate factors in role change is potentially difficult (Saxon, Gray and Oprescu, 2014; Noblet, Marriott and Rushton, 2019). Indeterminate factors have been described as those that are outside the rules and thus do not fit into the definition of a competency (Larson, 1977; Nancarrow, 2015), and which could be regarded as part of the artistry of a profession (Schon, 1992 in Kell and Owen, 2008). With a metric being the prescription writing rate and range (and with most of the participants prescribing on average under five scripts per week), making the case for additional physiotherapists to undertake the costly and time-consuming master’s level non-medical prescribing course was deemed difficult. Even though the participants were clear that becoming an independent prescriber had enabled them to deal more effectively with the complexities of primary care patients through a greater understanding of multi-pathology and poly-pharmacy, enhancing their practice particularly through conversations, and optimising progression rate and effectiveness of rehabilitation by understanding the patient’s general health status more, it was difficult to directly attribute this to the prescribing in a measurable way.

Establishing a profile for prescribing and creating a sustainable future pathway of prescribing development of staff and services emerged as a key concept. To address this, an individual and systems approach was suggested. Development and resourcing for individuals (resilience, self-efficacy, clinical mileage) and the healthcare system (promotion of physiotherapy independent prescribing and FCP roles beyond the physiotherapy profession, prescribing IT access, Controlled Drug legislation changes, evaluation evidence of prescribing specifically within FCP roles) are required. A potential vehicle for enabling support of this bilateral approach may be the establishment of prescribing communities of practice (Delgado *et al.*, 2020) to provide an environment that is safe, non-hierarchical and conducive to trusting communication, enabling sharing of vulnerability to be connective and generative and potentially reducing clinician’s sense of isolated responsibility (Delgado *et al.*, 2020). The importance of raising the profile of physiotherapists being able to independently prescribe to key stakeholder and commissioners was viewed as central to securing the long-term sustainability of prescribing physiotherapy MSk primary care services and aligned with Goodwin *et al.*’s (2020) findings in relation to the FCP role.

Sustainability of future generations of prescribers was also a key concern for the participants, recognising the risks of prescribing FCPs “asset stripping” traditional physiotherapy MSk services. The ‘First Contact Practitioners and Advanced Practitioners in Primary Care (Musculoskeletal): A Roadmap to Practice’ (HEE, 2020) should provide a robust educational framework to underpin the career development pathway to FCP roles and beyond into ACP. The requirement for independent prescribing within this ‘Roadmap to Practice’ framework is flexible, aligning with the ‘Musculoskeletal core capabilities framework for the first point of contact practitioners’ (Health Education England, NHS England and Skills for Health, 2018), which makes pharmacotherapy capability rather than independent prescribing a required capability. (Health Education England, NHS England and Skills for Health, 2018). Within MSk primary care, the physiotherapy accepted Controlled Drugs list was pivotal in undermining the physiotherapists’ sense of control, and participants were realistic in recognising that until this was resolved, there were significant frustrations impeding progress of the service and patient care, and personal self-efficacy of the prescribing physiotherapists involved.

Helping to drive and influence a public consultation review of permitted Controlled Drugs that physiotherapists can prescribe, notably Codeine, Gabapentin, and Pregabalin was a priority for this particular group of physiotherapy participants in this specific role and setting. Indeed, in October 2020, NHS England opened a “Consultation on proposed amendments to the list of Controlled Drugs that physiotherapists can independently prescribe across the United Kingdom” (NHS England, 2020), specifically noting a review of Codeine Phosphate, Tramadol Hydrochloride, Pregabalin, and Gabapentin. This consultation closed in December 2020 (NHS England, 2020), with the outcome awaited.

## Strengths of the research

The timing of the research was pertinent as just prior to the data collection, the NHS Long Term Plan (NHS England, 2019a) and specifically sweeping changes to primary care (NHS England, 2019b) were published, the physiotherapy accepted Controlled Drugs legislation changes were announced (CSP, 2019) and during the data collection phase, implemented. During write up, an increase from 70% to 100% funding for the physiotherapy FCP role in primary care as part of the funding for PCNs was announced (NHS England and British Medical Association (BMA), 2020) with prescribing supported as a potential postgraduate educational component of these roles for physiotherapists. This positioned the research in a contemporary setting and strengthened the rationale for investigation, at a micro level, of personal and professional experiences within the wider context.

## Limitations of the research

### Size of study

Whilst there were 15 participants from a range of professions and perspectives, interviewing more GPs may have provided some additional richness to the data.

### Range of participants

With a relatively small participant number, there was a risk of homogeneity of the participants or data collected. Patients were also not included as participants, although the importance of their experiences and views of primary care has been considered previously (Goodwin and Hendrick, 2016; Morris *et al.*, 2021), albeit not specific to prescribing. Increasing the range and number of participants further could have provided additional perspectives and potentially enhanced findings.

### Risk of researcher bias

As with all qualitative research, researcher bias and influence will exist. Participants were aware of me as a physiotherapy educator with an interest in the evolution of practice, and some of the language they used during interviews was subtly inclusive of me within the professional group. During all processes, I have endeavoured to address this overtly to represent the data and findings in as transparent a way as possible.

## Conclusion

By exploring the experience and practice of physiotherapy independent prescribing in primary care, this research addresses skills acquisition, changing scope of practice, ongoing competency and governance issues, autonomy, self-care, and sustainability. It enables learning from the early adopters of physiotherapy independent prescribing so that issues raised, and lessons learnt, can be considered and addressed in a timely manner to inform and support future developments both at individual and system levels.
